# Cholesterol Levels, Hormone Replacement Therapy, and Incident Dementia among Older Adult Women

**DOI:** 10.3390/nu15204481

**Published:** 2023-10-23

**Authors:** Huei-Ying Chiu, Hsin-Te Chang, Po-Chi Chan, Pai-Yi Chiu

**Affiliations:** 1 Department of Obstetrics and Gynecology, Show Chwan Memorial Hospital, Changhua 500, Taiwan; bobbie@docjames.net; 2 Department of Psychology, College of Science, Chung Yuan Christian University, Taoyuan 320, Taiwan; changht07182008@gmail.com; 3 Department of Neurology, Show Chwan Memorial Hospital, Changhua 500, Taiwan; nickchan16@gmail.com; 4 Department of Applied Mathematics, Tunghai University, Taichung 407, Taiwan

**Keywords:** cholesterol, incident dementia, Alzheimer’s disease, women, dementia

## Abstract

Previous studies revealed that hormone replacement therapy (HRT) probably has a protective effect for preventing dementia in post-menopausal women. However, the results were still controversial. The association between cholesterol levels and incident dementia in older women is not fully understood either. We conducted a retrospective analysis on a cohort of non-demented women aged older than 50 years, which was registered in the History-based Artificial Intelligence Clinical Dementia Diagnostic System database from September 2015 to August 2021. We followed this cohort longitudinally to examine the rates of conversion to dementia. Using a Cox regression model, we investigated the impact of the quartile of total cholesterol (TC) levels on incident dementia, adjusting for age, sex, education, neuropsychiatric symptoms, neuropsychological assessments, HRT, as well as various vascular risk factors and medications. We examined a cohort of 787 participants, comprising 539 (68.5%) individuals who did not develop dementia (non-converters). Among these non-converters, 68 individuals (12.6%) were treated with HRT. By contrast, there were 248 (31.5%) who did develop dementia (converters). Among the converters, 28 individuals (11.3%) were treated with HRT. The average follow-up durations were 2.9 ± 1.5 and 3.3 ± 1.6 years for non-converters and converters, respectively. Compared to the lowest quartile of TC levels (<153), the hazard ratios (HR) for converting to dementia were 0.61, 0.58, and 0.58 for the second (153–176), third (177–201), and highest (>201) quartiles, respectively (all *p* < 0.05). However, the low-density lipoprotein cholesterol (LDL-C) level and HRT did not alter the rate of conversion to dementia. In conclusion, the lowest quartile of TC increased incident dementia in post-menopausal women without dementia; however, HRT did not contribute to conversion to dementia. Some studies suggest that post-menopausal women who have reduced estrogen levels might have an increased risk of Alzheimer’s disease if they also have high cholesterol. Nonetheless, the evidence is inconclusive, as not all studies support this finding. The “Lower LDL-C is better” strategy for preventing cardiac vascular disease should be re-examined for the possible serial adverse effects of new onset dementia due to very low cholesterol levels.

## 1. Introduction

In recent times, dementia has been increasingly prevalent among the elderly, and this stems from various underlying causes. Notably, abnormal protein buildup has been found to coincide with compromised neurovasculature in individuals at different stages of dementia. The connection between vascular dysfunction and dementia was initially documented several decades ago [1]. Alzheimer’s disease (AD) stands as the prevailing variant of dementia and frequently manifests concurrently with vascular cognitive impairment (VCI) [2]. The neuropathological features characteristic of AD encompass the buildup of senile plaques, comprising an Aβ peptide, and the intraneuronal aggregation of neurofibrillary tangles (NFTs), composed of the hyperphosphorylated microtubule-binding protein tau (p-tau) [1]. Dementia with Lewy bodies ranks as the second most prevalent source of neurodegenerative dementia among older individuals [3]. Pathologically, DLB is identified by the anomalous accumulation of the synaptic protein α-synuclein, forming “Lewy bodies” within neurons, often accompanied by brain atrophy, albeit less pronounced than in AD [4].

There is evidence to suggest that hormonal changes during menopause may play a role in the development of dementia among post-menopausal women [5]. Estrogen is a hormone that declines sharply during menopause, and it is thought to have a protective effect on brain function [6]. Current hormone replacement therapy (HRT) encompasses conjugated equine estrogens (CEE), oral estradiol (E_2_) and transdermal estradiol, and esterified estrogen (EE) [7]. The typical initial oral doses for different estrogen formulations are as follows: 0.625 mg for CEE, 1 to 2 mg for E_2_, and 0.625 mg for EE. The standard dosage for transdermal E_2_ is 50 μg. It is essential to understand that the dosing schedules are established with consideration to biological endpoints and improvements in clinical symptoms [8]. There are two types of routes of administration for estrogen: oral and transdermal. Oral and transdermal estrogens reduce total cholesterol (TC) and low-density lipoprotein cholesterol (LDL-C). For post-menopausal women who have cardiovascular disease risk factors or are obese, the transdermal method of hormone therapy is the recommended choice [8]. Some studies have suggested that HRT with estrogen may reduce the risk of cognitive decline and dementia in post-menopausal women [9]. However, other studies have found conflicting results [10], and the use of HRT has been associated with increased risks of stroke, blood clots, and breast cancer [11,12,13], which yield increased risk of dementia. Therefore, the decision to use HRT should be made on an individual basis after weighing the potential benefits and risks. It is also important to note that there are other factors that may contribute to the development of dementia, such as genetics, lifestyle factors, and medical conditions. Therefore, further research is needed to fully understand the relationship between hormonal changes during menopause and the risk of dementia.

The association between low serum TC or LDL-C levels and the risk of dementia in older or post-menopausal women is not fully understood. While some studies have suggested that low cholesterol levels may be associated with an increased risk of dementia, others have found no significant association or have even suggested that high cholesterol levels or elevated levels of LDL-C, often referred to as “bad cholesterol”, may be a greater risk factor [14,15,16,17,18]. A plausible explanation for the association between low cholesterol levels and the risk of dementia is that cholesterol is important for the production and maintenance of cell membranes, including those in the brain [19,20,21]. The probable contributing factor for low cholesterol levels, inducing incident dementia, may include broken lipid rafts and demyelination in the brain [21,22,23]. Lowering TC or LDL-C levels may therefore be associated with impaired brain function and an increased risk of dementia [15,16,17]. One possible mechanism for the association of high LDL-C and dementia is that high levels of LDL-C can contribute to the formation of beta-amyloid plaques in the brain, which is a hallmark of AD [24,25]. Additionally, high cholesterol levels may lead to atherosclerosis and cause the narrowing of blood vessels, which can reduce blood flow to brain and impair cognitive function [26]. There is also evidence to suggest that treating high cholesterol with statin medications may reduce the risk of dementia in some individuals [27]. However, more research is needed to fully understand the relationship between cholesterol levels and the risk of dementia, as well as the potential benefits and risks of cholesterol-lowering medications in this context.

In this current study, based on the important physiological functions of cholesterol and controversial evidence of cholesterol levels and incident dementia, we hypothesized that sufficient serum cholesterol levels are essential for the maintenance of cognitive function, and cholesterol levels that are too low contribute to the development of dementia among older adults, especially post-menopausal women. Hence, in the clinical setting, the strategy of “lower LDL-C is better” for preventing cardiovascular disease should be reconsidered in light of the potential adverse effects of developing new-onset dementia as a result of very low cholesterol levels.

## 2. Materials and Methods

This retrospective study utilized data from the History-based Artificial Intelligence Clinical Dementia Diagnostic System (HAICDDS) project, which involved longitudinal follow-up. HAICDDS is presently employed to enroll dementia patients at four hospitals within the Show Chwan Healthcare System in Taiwan [28,29,30]. The primary objective of this study was to systematically and uniformly record individuals with cognitive impairment (CI) or dementia in this dataset. This dataset was intended for subsequent utilization in machine learning or deep learning in order to enhance the prediction, diagnosis, and differential diagnosis of stages and subtypes. Consequently, we enrolled a consecutive cohort of participants, which included both healthy individuals and patients exhibiting brain degeneration associated with AD or Lewy body disease (LBD), CI due to cerebrovascular disease (CVD), or other brain disorders associated with the decline of cognitive functions. Neuropsychologists who underwent comprehensive training conducted interviews with all participants and their informants. They instructed participants to undergo a battery of neuropsychological tests and complete surveys regarding their daily living activities. In addition to the Clinical Dementia Rating (CDR) scale [31], neuropsychiatric evaluations included the Cognitive Abilities Screening Instrument (CASI) [32] and the Montreal Cognitive Assessment (MoCA) [33]. We also evaluated the function of activities of daily living (ADL) using the History-based Artificial Intelligence Activities of Daily Living (HAIADL) [28] and assessed behavioral and psychological symptoms with the Neuropsychiatric Inventory (NPI) [34] to evaluate the severity of CI or dementia.

### 2.1. Diagnosis of Non-Dementia and Dementia in the HAICDDS Database

We defined non-dementia as follows: a global CDR [31] score of 0 or 0.5 with a CDR-SB < 4.5 [29,35,36,37]. Additionally, the CASI score was required to fall within the non-demented range after adjusting for sex, age, and education [32]. An HAIADL score < 8.5 were used to operationalize the absence of significant functional impairment [28]. We diagnosed dementia according to the criteria outlined by the National Institute on Aging-Alzheimer’s Association (NIA-AA) [38]. By contrast, participants were classified as having dementia if they exhibited impairments in two or more cognitive domains and experienced a decline in their daily functioning, indicated by a global CDR score ≥ 0.5 or a CDR-SB ≥ 4.5 [29,35,36,37]. We employed an HAIADL score > 8 to operationalize the diagnosis of functional impairment [28]. CI was defined using the CASI. The cutoff score, after adjusting for sex, age, and education, was required to fall within the range indicative of dementia [32].

### 2.2. Definition of Conversion

The initial evaluation, referred to as the baseline, was conducted for both converters (individuals who developed dementia) and non-converters (individuals who did not develop dementia). Subsequent follow-up tests were defined as checkpoints. Furthermore, at subsequent checkpoints, the scores for CDR, CDR-SB, MoCA, CASI, HAIADL, and IADL needed to demonstrate a decline compared to the baseline scores. For individuals who converted to dementia, the endpoint was determined by reaching the criteria for conversion without reverting to non-dementia stages. By contrast, for those who did not convert to dementia, the final checkpoint served as the endpoint.

### 2.3. Study Procedure

**Figure 1:**
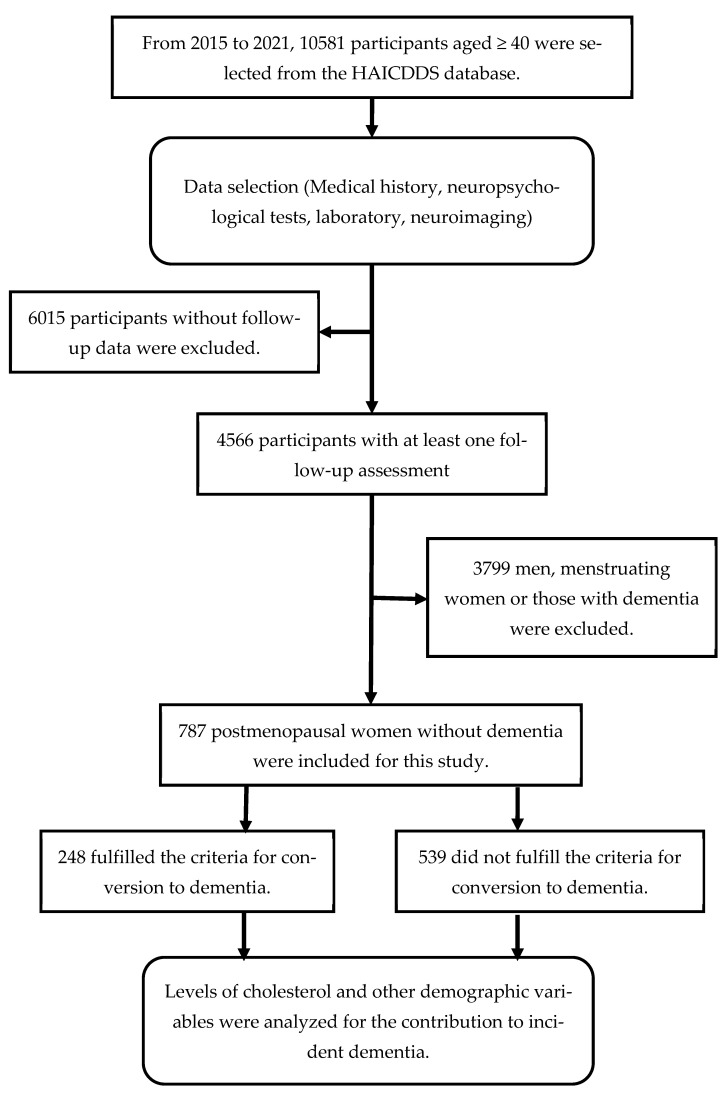
Flowchart illustrating participant selection and study procedure.

A comprehensive outline of the procedure is exhibited in Figure 1. Between September 2015 and December 2021, we recruited and analyzed individuals with a CDR < 1 who were older than 40 years and had undergone at least one follow-up assessment. Initially, a total of 10,581 participants were selected from the HAICDDS data. Participants without follow-up data, men, menstruating women, or those with dementia were excluded. Finally, 787 post-menopausal women without dementia were included for this study. The analysis encompassed the following datasets: (1) demographic information such as age, sex, education, duration of the follow-up, and a history of relevant medical conditions including dyslipidemia, cerebrovascular disease, hypertension, diabetes, congestive heart failure (CHF), carotid artery disease, and arrhythmia; and (2) the results of various assessments, including CDR-SB and neuropsychological tests such as MoCA, CASI, HAIADL, and NPI. Individuals who converted to dementia and those who did not were distinguished, and the hazard ratios (HRs) for the quartile of TC levels and demographic variables were analyzed. We also calculated and compared the conversion rates. 

### 2.4. Statistics

We utilized the Chinese version of IBM SPSS Statistics for Windows, version 22.0 (IBM Corp., Armonk, NY, USA), for conducting statistical analyses. Independent *t*-tests were employed to assess variables between two groups based on the assumption that two groups were independent from each other. Because our sample size was relatively large, the dependent variable was considered to be approximately normally distributed for each group. The homogeneity of variances between the two groups was assumed to be equal; this was checked using Levene’s test. These variables included age, education, follow-up duration, and the scores for CASI, CDR-SB, MoCA, HAIADL, and NPI. Lipid profiles, which encompassed TC, LDL-C, high-density lipoprotein cholesterol (HDL-C), triglyceride, and other measurable physical data were compared using independent *t*-tests. Meanwhile, sex distribution and the history of other relevant medical conditions were evaluated using the chi-square test. Analysis of HRT and other medications were performed by the chi-square test. We employed the Cox regression model within the non-demented cohort to explore the impact of TC or LDL-C levels on conversion to dementia. HRs were adjusted for all of the following confounding factors, including age, education, cognitive function (CASI), activities of daily living (HAIADL), neuropsychiatric symptoms (NPI), CVD, dyslipidemia, diabetes, hypertension, coronary artery disease (CAD), arrythmia, CHF, systolic blood pressure (SBP), fasting glucose, creatinine, and all medication. The conversion rates for various TC levels and all other factors were summarized. A significance level of *p* < 0.05 was applied to determine statistical significance for all analyses.

### 2.5. Ethical Consideration

This study was performed in a retrospective manner, with the data analyzed and processed in an anonymous fashion. The institutional review board of Show Chwan Memorial Hospital granted approval for this study and waived the need for informed consent (SCMH_IRB No: IRB1110503).

## 3. Results

A total of 787 participants were analyzed, including 539 (68.5%) non-converters and 248 (31.5%) converters with a mean follow-up of 2.9 ± 1.5 and 3.3 ± 1.6 years, respectively. In the comparison between the converter and non-converter groups before adjustment, the converter group had an older age, lower education, lower cognitive performance, lower daily functions, a history of diabetes, a history of using anti-diabetic drugs, and higher fasting glucose levels. Regular exercise, a history of hyperlipidemia, higher TC, and higher LDL-C decreased conversion rates (Table 1).

Table 2 demonstrates the results of the role of TC and other variables in the conversion from non-dementia to dementia among participants. HRs were compared with the non-converter group and were adjusted for several factors, including age, education, cognitive function (CASI), activities of daily living (HAIADL), neuropsychiatric symptoms (NPI), CVD, dyslipidemia, diabetes, hypertension, CAD, arrythmia, CHF, systolic blood pressure, fasting glucose, creatinine, and all medication. Compared to the non-converter group, the HRs for conversion to dementia, except for the contribution of TC levels, age (HR = 1.09; *p* < 0.001), education (HR = 1.09; *p* < 0.001), CASI score (HR = −0.98; *p* < 0.001), HAIADL score (HR = 1.11; *p* < 0.001), no exercise (HR = 1.27; *p* = 0.010), and SBP (HR = 1.01; *p* = 0.048), were also significantly associated with conversion. Worse neuropsychiatric symptoms showed a trend of increasing conversion rates (HR = 1.03; *p* = 0.057).

**Figure 2:**
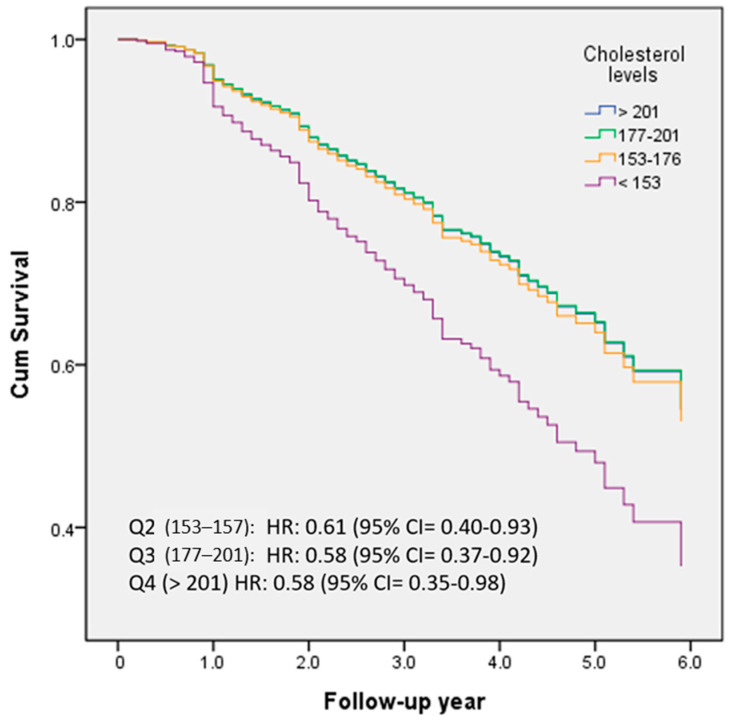
The percentage frequency of conversion was examined in relation to the quartiles of TC levels (Q1 < 153; Q2 153–176; Q3 177–201; Q4 > 201).

Figure 2 demonstrated the percentage frequency of conversion across different quartiles of TC levels (Q1 < 153; Q2 153–176; Q3 177–201; Q4 > 201). HRs were adjusted for age, education, follow-up years, cognitive function (CASI), activities of daily living (HAIADL), neuropsychiatric symptoms (NPI), dyslipidemia, diabetes, hypertension, CAD, atrial fibrillation, CHF, medications, and medical measurements. Compared to the lowest quartile (<153), the HRs for conversion to dementia were 0.61, 0.58, and 0.58 for the second (153–176), third (177–201), and highest (>201) quartiles, respectively (all *p* < 0.05). 

**Figure 3:**
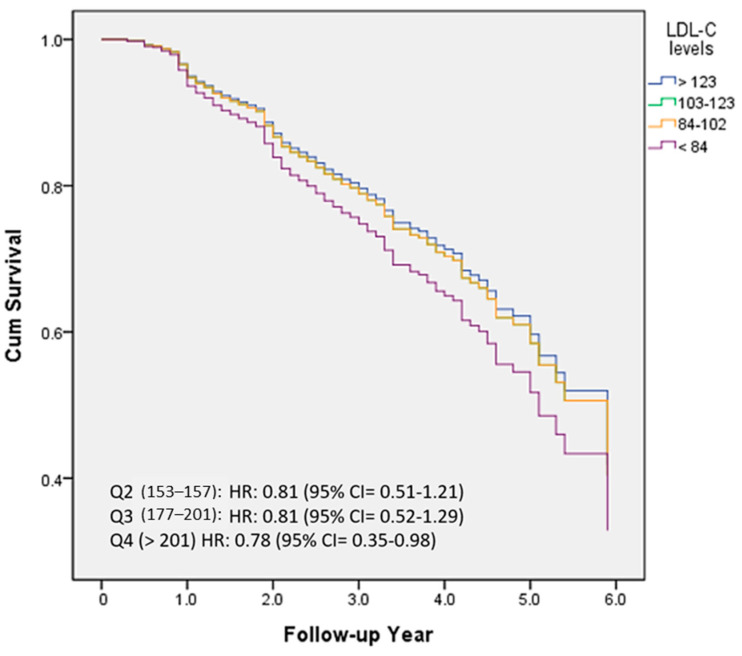
The percentage frequency of conversion according to the quartiles of LDL-C levels (Q1 < 84; Q2 84–102; Q3 103–123; Q4 > 123).

Figure 3 demonstrated the percentage frequency of conversion based on quartiles of LDL-C levels (Q1 < 84; Q2 84–102; Q3 103–123; Q4 > 123). HRs were adjusted for several variables, including age, education, follow-up years, cognitive function (CASI), activities of daily living (HAIADL), neuropsychiatric symptoms (NPI), dyslipidemia, diabetes, hypertension, CAD, atrial fibrillation, CHF, medications, and medical measurement. Compared to the Q1 group, HRs were 0.81, 0.81, and 0.78 in the Q2, Q3, and Q4 groups, respectively. However, no statistically significant differences were observed between these groups.

However, HRT did not alter the rate of conversion to dementia. Furthermore, older age, lower educational attainment, diminished cognitive performance, a history of diabetes, the use of anti-diabetic drugs, and higher fasting glucose levels increased conversion rates. On the other hand, regular exercise, a history of hyperlipidemia, higher TC, and higher LDL-C decreased conversion rates. 

## 4. Discussion

In this study, we applied relatively stringent criteria to determine dementia conversion. Conversion is defined as a decline in clinical assessment, encompassing the global stage, cognitive performance, and activities as assessed by CDR, CDR-SB, CASI, and HAIADL. A converter should demonstrate a decline in the global CDR stage without any returning to a higher level of cognitive performance or daily functioning throughout all follow-up assessments. 

The first aim of this study is to investigate the contribution of HRT; however, our result did not demonstrate significantly increased or decreased conversion rates in the non-demented female population. Moreover, a significant association of serum cholesterol levels, especially the TC level, was found to be associated with our population. The cohort with the lowest TC levels (TC < 153) in this study exhibited a significantly higher conversion rate to dementia. Compared to the lowest quartile, even the highest quartile group (TC > 201) showed significantly reduced conversion rates with an HR of 0.58 with 95% confidence interval = 0.35–0.98. We also saw a trend of similar inverse association of LDL-C levels with conversion rates; however, it did not reach clinical significance. By amalgamating these findings, our study could potentially offer additional evidence suggesting that TC is probably more important than LDL-C in predicting conversion to dementia in women without prior dementia. 

To explain the paradoxical association of TC or LDL-C levels with incident dementia, we considered the pathophysiology of the brain, including both lipid raft disruptions and demyelination, as being an important contribution factor to the clinical deterioration of the brain among people with low cholesterol levels. Firstly, lipid rafts are specialized regions in the cell membrane characterized by the dense packing of specific lipids and proteins [39,40,41]. In neurons, lipid rafts are also believed to be involved in synaptic function and plasticity, which are essential for learning and memory processes [42,43,44]. Low cholesterol caused by drugs or toxins may disrupt lipid rafts, subsequently affecting memory consolidation and cognitive function and finally resulting in dementia [45,46]. In addition to lipid raft disruption, demyelination caused by low cholesterol levels might also be another important factor that interferes with the regeneration of myeline; therefore, signal (information) transformation and consolidation become disrupted [47,48]. Several factors including hyperglycemia, hypertension, toxins, infections, and many other factors that induce free radicals, oxidation, and the inflammation of myeline results in the aging process or the destruction of myelin. In this situation, a higher cholesterol level might be a rate-limited process for repairment and remyelination [47,48]. Without intact functional lipid rafts and myeline, information in the brain for conduction, consolidation, or plasticity is not possible [45,46,47,48].

In our study population, several factors might also contribute to incident dementia in older women. Older age is a well-understandable risk factor for dementia. This finding is consistent with most of the previous studies [49,50]. A positive association of higher education levels with conversion rates in this study is not completely consistent with previous studies [49,50]. This discrepancy with previous studies may be attributed to differences in the time and geographical locations where the studies were conducted. Previous studies were conducted in earlier periods than ours and were carried out in the USA and Taiwan [49,50], likely in different cities compared to our study. In addition, baseline cognitive function, activities of daily living, and SBP all contributed to conversion to dementia, which were consistent with several previous studies [51,52]. 

Regarding sex differences in the risk of dementia, research indicates that women have a higher lifetime risk of developing AD compared to men [53]. However, these differences are multifaceted and might involve hormonal differences, differences in brain structure, and other factors [54]. The interplay between cholesterol levels and the risk of dementia in relation to sex differences has not been conclusively determined. Some studies have found that the relationship between high cholesterol levels and an increased risk of dementia might be more pronounced in women compared to men, especially during midlife [54]. Nevertheless, other studies have found no significant sex differences. Estrogen has been postulated to play a role in this association [55]. For instance, some studies suggest that post-menopausal women who have reduced estrogen levels might have a heightened risk of AD if they also have high cholesterol [56]. Nonetheless, the evidence is inconclusive, as not all studies support this finding [56].

Given that our study was conducted in Taiwan, it is important to note that our findings may not be applicable to broader populations. Factors related to Taiwan’s unique culture, dietary habits, or genetics could potentially impact the outcomes. Over 40% of dementia patients in Taiwan incorporated traditional Chinese medicine into their treatment [57]. Furthermore, a previous study provides evidence suggesting that the intake of fish, vegetables, tea, and coffee may hold potential benefits for mitigating dementia among the East Asian population [58]. An investigation of cerebral autosomal dominant arteriopathy with subcortical infarcts and leukoencephalopathy in the Taiwanese population revealed that individuals with *NOTCH3* mutations exhibited a greater prevalence of cerebral microbleeds in regions such as the thalamus and temporal lobe, which is associated with dementia [59].

There are several limitations of this study that need to be addressed. First, although medication for hormone and lipid lowering were adjusted in this study, the detailed medication types and doses did not undergo separate analysis for their detailed contribution due to the relatively small sample size. Second, the research took place in three different centers in Taiwan. Further studies involving a broader range of centers across various countries or ethnicities to explore the role of TC or LDL-C in tracking conversion rates in pre-demented women are warranted. Third, the follow-up periods of both non-converter and converter groups were relatively short, and these were 2.9 ± 1.5 and 3.3 ± 1.6 years, respectively. Fourth, although this study followed a longitudinal approach, the design was retrospective and not preplanned, which may have introduced some imprecision in the data. Consequently, there is a need for a more rigorous prospective longitudinal study with a larger sample size. 

In conclusion, the lowest quartile of TC (<153) increased incident dementia in post-menopausal women without dementia; however, LDL-C levels or lipid-lowering drugs did not influence conversion rates. The “Lower LDL-C is better” strategy for the prevention of cardiac vascular disease should be re-examined for the possible serial adverse effects of new-onset dementia due to very low cholesterol levels.

## Figures and Tables

**Table 1:** Comparison of demographical information among participants without dementia, stratified into the non-converter and converter groups.

	Non-Converters Mean (SD)	Converters Mean (SD)	*p*-Value
N	539	248	
Age, year	70.3 (8.8)	77.0 (7.2)	<0.001
Education, year	5.2 (4.4)	4.1 (3.8)	<0.001
Follow-up, year	2.9 (1.5)	3.3 (1.6)	<0.001
CDR-SB	1.3 (1.1)	2.2 (1.2)	<0.001
CASI	76.2 (12.4)	67.7 (11.1)	<0.001
MoCA	17.2 (6.4)	12.6 (4.9)	<0.001
HAIADL	3.2 (2.4)	5.0 (2.8)	<0.001
NPI	4.4 (6.3)	4.6 (5.5)	NS
Cerebrovascular disease, N (%)	106 (19.7)	64 (25.8)	NS
Hypertension, N (%)	378 (70.1)	189 (76.2)	NS
Diabetes, N (%)	192 (35.6)	108 (43.5)	0.040
Dyslipidemia, N (%)	348 (64.6)	133 (53.6)	0.005
Coronary artery disease, N (%)	52 (9.6)	33 (13.3)	NS
Atrial fibrillation, N (%)	70 (13.0)	48 (19.4)	0.024
Congestive heart failure, N (%)	42 (7.8)	32 (12.9)	0.026
Regular exercise, N (%)	288 (53.4)	83 (33.5)	<0.001
Hormone replacement therapy, N (%)	68 (12.6)	28 (11.3)	NS
Anti-Hypertensive, N (%)	378 (70.1)	189 (76.2)	NS
Anti-Diabetes, N (%)	192 (35.6)	108 (43.5)	0.021
Lipid-lowering drugs, N (%)	348 (64.6)	133 (53.6)	NS
Total Cholesterol	182.9 (35.8)	170.5 (39.6)	<0.001
Low-density lipoprotein cholesterol	109.4 (32.2)	98.8 (33.1)	<0.001
High-density lipoprotein cholesterol	54.8 (15.1)	53.4 (17.4)	NS
Triglyceride	129.7 (80.1)	129.8 (87.2)	NS
Systolic blood pressure	134.0 (18.3)	135.9 (18.2)	NS
Ac glucose	112.6 (36.0)	119.0 (41.3)	0.047
Body mass index	24.8 (3.1)	24.0 (3.7)	NS
Creatinine	0.8 (0.4)	1.0 (0.6)	0.002

CDR-SB, sum of the boxes of the Clinical Dementia Rating scale; N, number; SD, Standard deviation; NS, non-significance; CASI, Cognitive Abilities Screening Instrument; MoCA, Montreal Cognitive Assessment; IADL, Activities of Daily Living in the History-based Artificial Intelligence Clinical Diagnosis of Dementia System; NPI, Neuropsychiatric Inventory.

**Table 2:** The impact of TC and various other variables on the conversion from non-dementia to dementia in the participants of this study. HRs were compared with the non-converter group and adjusted for factors including age, education, cognitive function (CASI), activities of daily living (HAIADL), neuropsychiatric symptoms (NPI), CVD, dyslipidemia, diabetes, hypertension, CAD, arrythmia, CHF, SBP, fasting glucose, creatinine, and all medication.

Variables	B	Wald	Sig	Exp	95% Confidence Interval for Exp
Cholesterol					
Q1 (<153)	0				
Q2 (153–176)	−0.54	4.10	0.043	0.58	0.35–0.98
Q3 (177–201)	−0.55	5.39	0.020	0.58	0.37–0.92
Q4 (>201)	−0.50	5.28	0.022	0.61	0.70–0.93
Age	0.07	30.42	<0.001	1.09	1.05–1.10
Education	0.09	12.28	<0.001	1.09	1.04–1.14
CASI	−0.03	6.74	0.009	0.98	0.96–0.99
HAIADL	0.11	10.64	0.001	1.11	1.04–1.19
NPI	0.03	3.62	0.057	1.03	1.00–1.05
CVD	0.14	0.53	0.465	1.15	0.79–1.67
diabetes	−0.00	0.00	0.996	1.00	0.52–1.93
hypertension	0.06	0.02	0.888	1.06	0.49–2.27
dyslipidemia	−0.01	0.00	0.973	0.99	0.65–1.52
CAD	0.32	1.89	0.169	1.38	0.87–2.17
CHD	0.40	2.66	0.103	1.49	0.92–2.42
No exercise	0.24	6.66	0.010	1.27	1.06–1.53
Hormone replacement therapy	0.24	0.79	0.375	1.26	0.75–2.13
Antihypertensives	−0.04	0.01	0.928	0.97	0.45–2.06
Anti-diabetes	0.09	0.06	0.803	1.09	0.55–2.17
Lipid-lowering drugs	−0.15	0.52	0.470	0.86	0.58–1.29
SBP	0.01	0.39	0.048	1.01	1.00–1.02
Fasting glucose	0.00	0.53	0.468	1.00	1.00–1.01
Creatinine	0.11	0.67	0.413	1.12	0.86–1.46

CASI: Cognitive Abilities Screening Instrument; HAIADL: History-based Artificial Intelligence Activities of Daily Living; NPI: Neuropsychiatric Inventory.

## Data Availability

All data and material are included in the manuscript.

## References

[1] Raz L., Knoefel J., Bhaskar K. (2016). The neuropathology and cerebrovascular mechanisms of dementia. J. Cereb. Blood Flow Metab..

[2] Hyman B.T., Phelps C.H., Beach T.G., Bigio E.H., Cairns N.J., Carrillo M.C., Dickson D.W., Duyckaerts C., Frosch M.P., Masliah E. (2012). National Institute on Aging-Alzheimer’s Association guidelines for the neuropathologic assessment of Alzheimer’s disease. Alzheimers. Dement..

[3] Barker W.W., Luis C.A., Kashuba A., Luis M., Harwood D.G., Loewenstein D., Waters C., Jimison P., Shepherd E., Sevush S. (2002). Relative frequencies of Alzheimer disease, Lewy body, vascular and frontotemporal dementia, and hippocampal sclerosis in the State of Florida Brain Bank. Alzheimer Dis. Assoc. Disord..

[4] Spillantini M.G., Schmidt M.L., Lee V.M., Trojanowski J.Q., Jakes R., Goedert M. (1997). Alpha-synuclein in Lewy bodies. Nature.

[5] Pertesi S., Coughlan G., Puthusseryppady V., Morris E., Hornberger M. (2019). Menopause, cognition and dementia—A review. Post Reprod. Health.

[6] Hogervorst E., Bandelow S. (2009). Brain and cognition. Is there any case for improving cognitive function in menopausal women using estrogen treatment?. Minerva Ginecol..

[7] Langer R.D., Hodis H.N., Lobo R.A., Allison M.A. (2021). Hormone replacement therapy—Where are we now?. Climacteric.

[8] Flores V.A., Pal L., Manson J.E. (2021). Hormone Therapy in Menopause: Concepts, Controversies, and Approach to Treatment. Endocr. Rev..

[9] Stute P., Wienges J., Koller A.S., Giese C., Wesemuller W., Janka H., Baumgartner S. (2021). Cognitive health after menopause: Does menopausal hormone therapy affect it?. Best Pract. Res. Clin. Endocrinol. Metab..

[10] Pourhadi N., Morch L.S., Holm E.A., Torp-Pedersen C., Meaidi A. (2023). Menopausal hormone therapy and dementia: Nationwide, nested case-control study. BMJ.

[11] Jones M.E., Schoemaker M.J., Wright L., McFadden E., Griffin J., Thomas D., Hemming J., Wright K., Ashworth A., Swerdlow A.J. (2016). Menopausal hormone therapy and breast cancer: What is the true size of the increased risk?. Br. J. Cancer.

[12] Johansson T., Fowler P., Ek W.E., Skalkidou A., Karlsson T., Johansson A. (2022). Oral Contraceptives, Hormone Replacement Therapy, and Stroke Risk. Stroke.

[13] Strothmann A., Schneider H.P. (2003). Hormone therapy: The European women’s perspective. Climacteric.

[14] Solomon A., Kivipelto M., Wolozin B., Zhou J., Whitmer R.A. (2009). Midlife serum cholesterol and increased risk of Alzheimer’s and vascular dementia three decades later. Dement. Geriatr. Cogn. Disord..

[15] Stewart R., White L.R., Xue Q.L., Launer L.J. (2007). Twenty-six-year change in total cholesterol levels and incident dementia: The Honolulu-Asia Aging Study. Arch. Neurol..

[16] Mielke M.M., Zandi P.P., Sjogren M., Gustafson D., Ostling S., Steen B., Skoog I. (2005). High total cholesterol levels in late life associated with a reduced risk of dementia. Neurology.

[17] Ding D., Zhou F., Cao Y., Liang X., Wu W., Xiao Z., Zhao Q., Deng W. (2021). Cholesterol profiles and incident cognitive decline among older adults: The Shanghai Aging Study. Age Ageing.

[18] Moroney J.T., Tang M.X., Berglund L., Small S., Merchant C., Bell K., Stern Y., Mayeux R. (1999). Low-density lipoprotein cholesterol and the risk of dementia with stroke. JAMA.

[19] Marin R., Fabelo N., Martin V., Garcia-Esparcia P., Ferrer I., Quinto-Alemany D., Diaz M. (2017). Anomalies occurring in lipid profiles and protein distribution in frontal cortex lipid rafts in dementia with Lewy bodies disclose neurochemical traits partially shared by Alzheimer’s and Parkinson’s diseases. Neurobiol. Aging.

[20] Reitz C. (2012). Dyslipidemia and dementia: Current epidemiology, genetic evidence, and mechanisms behind the associations. J. Alzheimers Dis..

[21] Schengrund C.L. (2010). Lipid rafts: Keys to neurodegeneration. Brain Res. Bull..

[22] Michel V., Bakovic M. (2007). Lipid rafts in health and disease. Biol. Cell.

[23] Moll T., Marshall J.N.G., Soni N., Zhang S., Cooper-Knock J., Shaw P.J. (2021). Membrane lipid raft homeostasis is directly linked to neurodegeneration. Essays Biochem..

[24] Agarwal M., Khan S. (2020). Plasma Lipids as Biomarkers for Alzheimer’s Disease: A Systematic Review. Cureus.

[25] Suryadevara V., Storey S.G., Aronow W.S., Ahn C. (2003). Association of abnormal serum lipids in elderly persons with atherosclerotic vascular disease and dementia, atherosclerotic vascular disease without dementia, dementia without atherosclerotic vascular disease, and no dementia or atherosclerotic vascular disease. J. Gerontol. A Biol. Sci. Med. Sci..

[26] Ban Y., Watanabe T., Miyazaki A., Nakano Y., Tobe T., Idei T., Iguchi T., Ban Y., Katagiri T. (2007). Impact of increased plasma serotonin levels and carotid atherosclerosis on vascular dementia. Atherosclerosis.

[27] Zandi P.P., Sparks D.L., Khachaturian A.S., Tschanz J., Norton M., Steinberg M., Welsh-Bohmer K.A., Breitner J.C., Cache County Study Investigators (2005). Do statins reduce risk of incident dementia and Alzheimer disease? The Cache County Study. Arch. Gen. Psychiatry.

[28] Hung C.H., Hung G.U., Wei C.Y., Tzeng R.C., Chiu P.Y. (2021). Function-based dementia severity assessment for vascular cognitive impairment. J. Formos. Med. Assoc..

[29] Yang Y.W., Hsu K.C., Wei C.Y., Tzeng R.C., Chiu P.Y. (2021). Operational Determination of Subjective Cognitive Decline, Mild Cognitive Impairment, and Dementia Using Sum of Boxes of the Clinical Dementia Rating Scale. Front. Aging Neurosci..

[30] Wang T.Y., Chang W.L., Wei C.Y., Liu C.H., Tzeng R.C., Chiu P.Y. (2023). Cholesterol Paradox in Older People with Type 2 Diabetes Mellitus Regardless of Lipid-Lowering Drug Use: A Cross-Sectional Cohort Study. Nutrients.

[31] Morris J.C. (1993). The Clinical Dementia Rating (CDR): Current version and scoring rules. Neurology.

[32] Lin K.N., Wang P.N., Liu C.Y., Chen W.T., Lee Y.C., Liu H.C. (2002). Cutoff scores of the cognitive abilities screening instrument, Chinese version in screening of dementia. Dement. Geriatr. Cogn. Disord..

[33] Nasreddine Z.S., Phillips N.A., Bedirian V., Charbonneau S., Whitehead V., Collin I., Cummings J.L., Chertkow H. (2005). The Montreal Cognitive Assessment, MoCA: A brief screening tool for mild cognitive impairment. J. Am. Geriatr. Soc..

[34] Cummings J.L. (1997). The Neuropsychiatric Inventory: Assessing psychopathology in dementia patients. Neurology.

[35] O’Bryant S.E., Lacritz L.H., Hall J., Waring S.C., Chan W., Khodr Z.G., Massman P.J., Hobson V., Cullum C.M. (2010). Validation of the new interpretive guidelines for the clinical dementia rating scale sum of boxes score in the national Alzheimer’s coordinating center database. Arch. Neurol..

[36] O’Bryant S.E., Waring S.C., Cullum C.M., Hall J., Lacritz L., Massman P.J., Lupo P.J., Reisch J.S., Doody R., Texas Alzheimer’s Research C. (2008). Staging dementia using Clinical Dementia Rating Scale Sum of Boxes scores: A Texas Alzheimer’s research consortium study. Arch. Neurol..

[37] Tzeng R.C., Yang Y.W., Hsu K.C., Chang H.T., Chiu P.Y. (2022). Sum of boxes of the clinical dementia rating scale highly predicts conversion or reversion in predementia stages. Front. Aging Neurosci..

[38] McKhann G.M., Knopman D.S., Chertkow H., Hyman B.T., Jack C.R., Kawas C.H., Klunk W.E., Koroshetz W.J., Manly J.J., Mayeux R. (2011). The diagnosis of dementia due to Alzheimer’s disease: Recommendations from the National Institute on Aging-Alzheimer’s Association workgroups on diagnostic guidelines for Alzheimer’s disease. Alzheimers Dement..

[39] Simons K., Toomre D. (2000). Lipid rafts and signal transduction. Nat. Rev. Mol. Cell Biol..

[40] Jacobson K., Mouritsen O.G., Anderson R.G. (2007). Lipid rafts: At a crossroad between cell biology and physics. Nat. Cell Biol..

[41] Lingwood D., Simons K. (2010). Lipid rafts as a membrane-organizing principle. Science.

[42] Tsui-Pierchala B.A., Encinas M., Milbrandt J., Johnson E.M. (2002). Lipid rafts in neuronal signaling and function. Trends Neurosci..

[43] Lai A.Y., McLaurin J. (2010). Mechanisms of amyloid-Beta Peptide uptake by neurons: The role of lipid rafts and lipid raft-associated proteins. Int. J. Alzheimers Dis..

[44] Igarashi M., Honda A., Kawasaki A., Nozumi M. (2020). Neuronal Signaling Involved in Neuronal Polarization and Growth: Lipid Rafts and Phosphorylation. Front. Mol. Neurosci..

[45] Frank C., Rufini S., Tancredi V., Forcina R., Grossi D., D’Arcangelo G. (2008). Cholesterol depletion inhibits synaptic transmission and synaptic plasticity in rat hippocampus. Exp. Neurol..

[46] Krivoi I.I., Petrov A.M. (2019). Cholesterol and the Safety Factor for Neuromuscular Transmission. Int. J. Mol. Sci..

[47] Saher G., Brugger B., Lappe-Siefke C., Mobius W., Tozawa R., Wehr M.C., Wieland F., Ishibashi S., Nave K.A. (2005). High cholesterol level is essential for myelin membrane growth. Nat. Neurosci..

[48] Berghoff S.A., Spieth L., Sun T., Hosang L., Depp C., Sasmita A.O., Vasileva M.H., Scholz P., Zhao Y., Krueger-Burg D. (2021). Neuronal cholesterol synthesis is essential for repair of chronically demyelinated lesions in mice. Cell. Rep..

[49] Langa K.M., Larson E.B., Crimmins E.M., Faul J.D., Levine D.A., Kabeto M.U., Weir D.R. (2017). A Comparison of the Prevalence of Dementia in the United States in 2000 and 2012. JAMA Intern. Med..

[50] Sun Y., Lee H.J., Yang S.C., Chen T.F., Lin K.N., Lin C.C., Wang P.N., Tang L.Y., Chiu M.J. (2014). A nationwide survey of mild cognitive impairment and dementia, including very mild dementia, in Taiwan. PLoS ONE.

[51] Gray S.L., Anderson M.L., Hubbard R.A., LaCroix A., Crane P.K., McCormick W., Bowen J.D., McCurry S.M., Larson E.B. (2013). Frailty and incident dementia. J. Gerontol. A Biol. Sci. Med. Sci..

[52] Stewart R., Xue Q.L., Masaki K., Petrovitch H., Ross G.W., White L.R., Launer L.J. (2009). Change in blood pressure and incident dementia: A 32-year prospective study. Hypertension.

[53] Andersen K., Launer L.J., Dewey M.E., Letenneur L., Ott A., Copeland J.R., Dartigues J.F., Kragh-Sorensen P., Baldereschi M., Brayne C. (1999). Gender differences in the incidence of AD and vascular dementia: The EURODEM Studies. EURODEM Incidence Research Group. Neurology.

[54] Azad N.A., Al Bugami M., Loy-English I. (2007). Gender differences in dementia risk factors. Gend. Med..

[55] Dufouil C., Richard F., Fievet N., Dartigues J.F., Ritchie K., Tzourio C., Amouyel P., Alperovitch A. (2005). APOE genotype, cholesterol level, lipid-lowering treatment, and dementia: The Three-City Study. Neurology.

[56] Hogervorst E., Williams J., Budge M., Riedel W., Jolles J. (2000). The nature of the effect of female gonadal hormone replacement therapy on cognitive function in post-menopausal women: A meta-analysis. Neuroscience.

[57] Lin S.K., Tsai Y.T., Lai J.N., Wu C.T. (2015). Demographic and medication characteristics of traditional Chinese medicine users among dementia patients in Taiwan: A nationwide database study. J. Ethnopharmacol..

[58] Lee C.Y., Sun Y., Lee H.J., Chen T.F., Wang P.N., Lin K.N., Tang L.Y., Lin C.C., Chiu M.J. (2017). Modest Overweight and Healthy Dietary Habits Reduce Risk of Dementia: A Nationwide Survey in Taiwan. J. Prev. Alzheimers Dis..

[59] Ho W.M., Wu Y.Y., Chen Y.C. (2020). Genetic Variants behind Cardiovascular Diseases and Dementia. Genes.

